# Finding Our Way through Phenotypes

**DOI:** 10.1371/journal.pbio.1002033

**Published:** 2015-01-06

**Authors:** Andrew R. Deans, Suzanna E. Lewis, Eva Huala, Salvatore S. Anzaldo, Michael Ashburner, James P. Balhoff, David C. Blackburn, Judith A. Blake, J. Gordon Burleigh, Bruno Chanet, Laurel D. Cooper, Mélanie Courtot, Sándor Csösz, Hong Cui, Wasila Dahdul, Sandip Das, T. Alexander Dececchi, Agnes Dettai, Rui Diogo, Robert E. Druzinsky, Michel Dumontier, Nico M. Franz, Frank Friedrich, George V. Gkoutos, Melissa Haendel, Luke J. Harmon, Terry F. Hayamizu, Yongqun He, Heather M. Hines, Nizar Ibrahim, Laura M. Jackson, Pankaj Jaiswal, Christina James-Zorn, Sebastian Köhler, Guillaume Lecointre, Hilmar Lapp, Carolyn J. Lawrence, Nicolas Le Novère, John G. Lundberg, James Macklin, Austin R. Mast, Peter E. Midford, István Mikó, Christopher J. Mungall, Anika Oellrich, David Osumi-Sutherland, Helen Parkinson, Martín J. Ramírez, Stefan Richter, Peter N. Robinson, Alan Ruttenberg, Katja S. Schulz, Erik Segerdell, Katja C. Seltmann, Michael J. Sharkey, Aaron D. Smith, Barry Smith, Chelsea D. Specht, R. Burke Squires, Robert W. Thacker, Anne Thessen, Jose Fernandez-Triana, Mauno Vihinen, Peter D. Vize, Lars Vogt, Christine E. Wall, Ramona L. Walls, Monte Westerfeld, Robert A. Wharton, Christian S. Wirkner, James B. Woolley, Matthew J. Yoder, Aaron M. Zorn, Paula Mabee

**Affiliations:** 1 Department of Entomology, Pennsylvania State University, University Park, Pennsylvania, United States of America; 2 Genome Division, Lawrence Berkeley National Lab, Berkeley, California, United States of America; 3 Department of Plant Biology, Carnegie Institution for Science, Stanford, California, United States of America; 4 Phoenix Bioinformatics, Palo Alto, California, United States of America; 5 School of Life Sciences, Arizona State University, Tempe, Arizona, United States of America; 6 Department of Genetics, University of Cambridge, Cambridge, United Kingdom; 7 National Evolutionary Synthesis Center, Durham, North Carolina, United States of America; 8 Department of Vertebrate Zoology and Anthropology, California Academy of Sciences, San Francisco, California, United States of America; 9 The Jackson Laboratory, Bar Harbor, Maine, United States of America; 10 Department of Biology, University of Florida, Gainesville, Florida, United States of America; 11 Muséum national d'Histoire naturelle, Département Systématique et Evolution, Paris, France; 12 Department of Botany and Plant Pathology, Oregon State University, Corvallis, Oregon, United States of America; 13 Molecular Biology and Biochemistry Department, Simon Fraser University, Burnaby, British Columbia, Canada; 14 MTA-ELTE-MTM, Ecology Research Group, Pázmány Péter sétány 1C, Budapest, Hungary; 15 School of Information Resources and Library Science, University of Arizona, Tucson, Arizona, United States of America; 16 Department of Biology, University of South Dakota, Vermillion, South Dakota, United States of America; 17 Department of Botany, University of Delhi, Delhi, India; 18 Department of Anatomy, Howard University College of Medicine, Washington D.C., United States of America; 19 Department of Oral Biology, College of Dentistry, University of Illinois, Chicago, Illinois, United States of America; 20 Stanford Center for Biomedical Informatics Research, Stanford, California, United States of America; 21 Biocenter Grindel and Zoological Museum, Hamburg University, Hamburg, Germany; 22 Department of Computer Science, Aberystwyth University, Aberystwyth, Ceredigion, United Kingdom; 23 Department of Medical Informatics & Epidemiology, Oregon Health & Science University, Portland, Oregon, United States of America; 24 Department of Biological Sciences, University of Idaho, Moscow, Idaho, United States of America; 25 Mouse Genome Informatics, The Jackson Laboratory, Bar Harbor, Maine, United States of America; 26 Unit for Laboratory Animal Medicine, Department of Microbiology and Immunology, Center for Computational Medicine and Bioinformatics, and Comprehensive Cancer Center, University of Michigan Medical School, Ann Arbor, Michigan, United States of America; 27 Department of Organismal Biology and Anatomy, University of Chicago, Chicago, Illinois, United States of America; 28 Cincinnati Children's Hospital, Division of Developmental Biology, Cincinnati, Ohio, United States of America; 29 Institute for Medical Genetics and Human Genetics, Charité-Universitätsmedizin Berlin, Berlin, Germany; 30 Department of Genetics, Development and Cell Biology and Department of Agronomy, Iowa State University, Ames, Iowa, United States of America; 31 Signalling ISP, Babraham Institute, Babraham, Cambridgeshire, UK; 32 Department of Ichthyology, The Academy of Natural Sciences, Philadelphia, Pennsylvania, United States of America; 33 Eastern Cereal and Oilseed Research Centre, Ottawa, Ontario, Canada; 34 Department of Biological Science, Florida State University, Tallahassee, Florida, United States of America; 35 Richmond, Virginia, United States of America; 36 European Molecular Biology Laboratory - European Bioinformatics Institute, Wellcome Trust Genome Campus, Hinxton, United Kingdom; 37 Division of Arachnology, Museo Argentino de Ciencias Naturales - CONICET, Buenos Aires, Argentina; 38 Allgemeine & Spezielle Zoologie, Institut für Biowissenschaften, Universität Rostock, Universitätsplatz 2, Rostock, Germany; 39 Institut für Medizinische Genetik und Humangenetik Charité – Universitätsmedizin Berlin, Berlin, Germany; 40 School of Dental Medicine, University at Buffalo, Buffalo, New York, United States of America; 41 Smithsonian Institution, National Museum of Natural History, Washington, D.C., United States of America; 42 Knight Cancer Institute, Oregon Health & Science University, Portland, Oregon, United States of America; 43 Division of Invertebrate Zoology, American Museum of Natural History, New York, New York, United States of America; 44 Department of Entomology, University of Kentucky, Lexington, Kentucky, United States of America; 45 Department of Biological Sciences, Northern Arizona University, Flagstaff, Arizona, United States of America; 46 Department of Philosophy, University at Buffalo, Buffalo, New York, United States of America; 47 Department of Plant and Microbial Biology, Integrative Biology, and the University and Jepson Herbaria, University of California, Berkeley, California, United States of America; 48 Bioinformatics and Computational Biosciences Branch, Office of Cyber Infrastructure and Computational Biology, National Institute of Allergy and Infectious Diseases, National Institutes of Health, Bethesda, Maryland, United States of America; 49 Department of Biology, University of Alabama at Birmingham, Birmingham, Alabama, United States of America; 50 The Data Detektiv, 1412 Stearns Hill Road, Waltham, Massachusetts, United States of America; 51 Canadian National Collection of Insects, Ottawa, Ontario, Canada; 52 Department of Experimental Medical Science, Lund University, Lund, Sweden; 53 Department of Biological Sciences, University of Calgary, Calgary, Alberta, Canada; 54 Universität Bonn, Institut für Evolutionsbiologie und Ökologie, Bonn, Germany; 55 Department of Evolutionary Anthropology, Duke University, Durham, North Carolina, United States of America; 56 iPlant Collaborative University of Arizona, Thomas J. Keating Bioresearch Building, Tucson, Arizona, United States of America; 57 Institute of Neuroscience, University of Oregon, Eugene, Oregon, United States of America; 58 Department of Entomology, Texas A & M University, College, Station, Texas, United States of America; 59 Illinois Natural History Survey, University of Illinois, Champaign, Illinois, United States of America

## Abstract

Despite a large and multifaceted effort to understand the vast landscape of phenotypic data, their current form inhibits productive data analysis. The lack of a community-wide, consensus-based, human- and machine-interpretable language for describing phenotypes and their genomic and environmental contexts is perhaps the most pressing scientific bottleneck to integration across many key fields in biology, including genomics, systems biology, development, medicine, evolution, ecology, and systematics. Here we survey the current phenomics landscape, including data resources and handling, and the progress that has been made to accurately capture relevant data descriptions for phenotypes. We present an example of the kind of integration across domains that computable phenotypes would enable, and we call upon the broader biology community, publishers, and relevant funding agencies to support efforts to surmount today's data barriers and facilitate analytical reproducibility.

## Abstract 2

Imagine if we could compute across phenotype data as easily as genomic data; this article calls for efforts to realize this vision and discusses the potential benefits.

## Introduction

**Figure 1:**
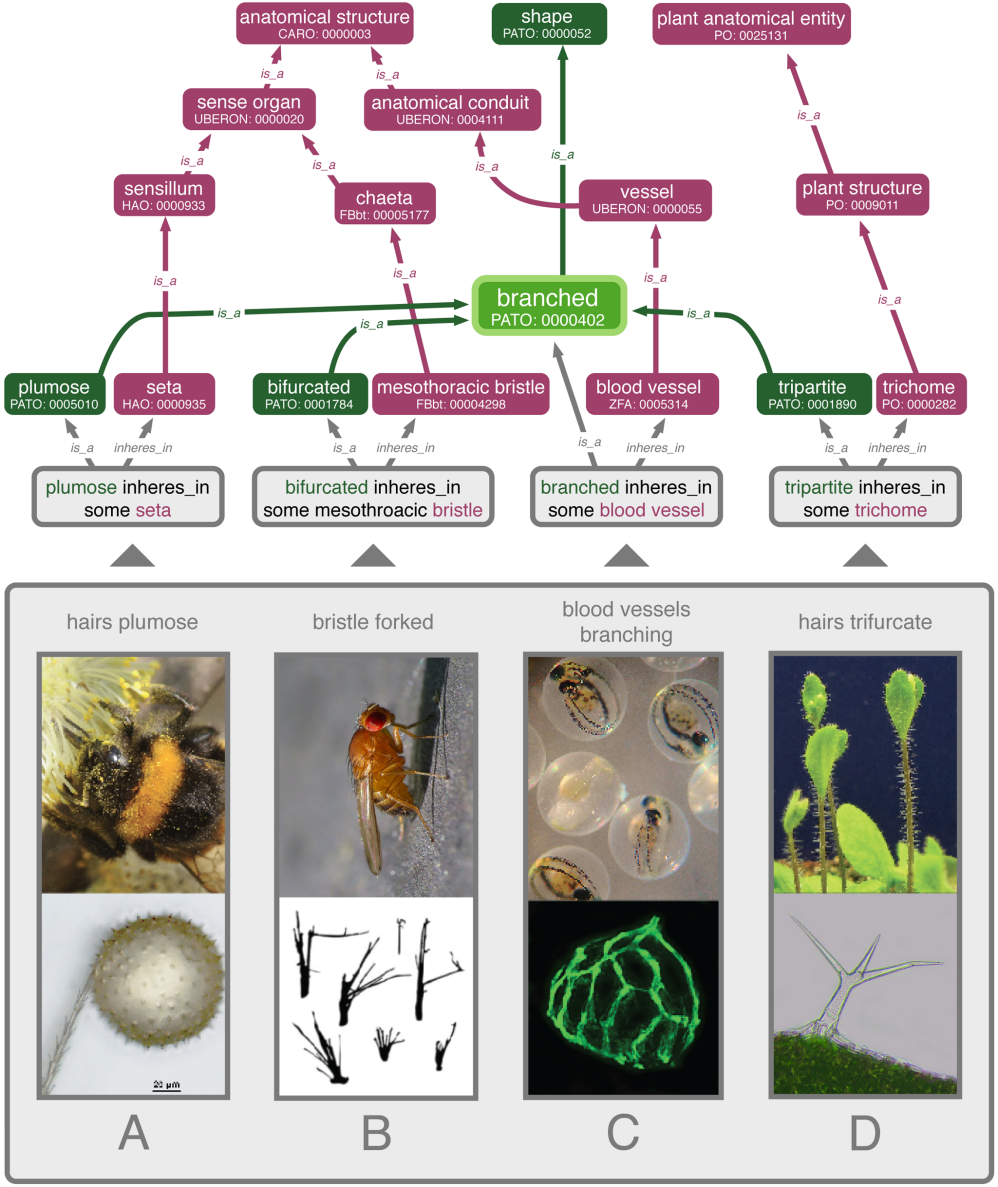
How to discover branching phenotypes? (Bottom panel) Phenotype data exhibiting various forms of branchiness are not easily discerned from diverse natural language descriptions. (A) Bee hairs are different from most other insect hairs in that they are plumose, which facilitates pollen collection. (B) A mutant of *Drosophila melanogaster* exhibits forked bristles, due to a variation in *mical*. (C) In zebrafish larvae (*Danio rerio*), angiogenesis begins with vessels branching. (D) Plant trichomes take on many forms, including trifurcation. (Top) Phenotypes involving some type of “branched” are easily recovered when they are represented with ontologies. In a semantic graph, free text descriptions are converted into phenotype statements involving an anatomy term from animal or plant ontologies [56],[118] and a quality term from a quality ontology [106], connected by a logical expression (“inheres_in some”). Anatomy (purple) and quality (green) terms (ontology IDs beneath) relate phenotype statements from different species by virtue of the logic inherent in the ontologies, e.g., plumose, bifurcated, branched, and tripartite are all subtypes of “branched.” Image credits: bumble bee with pollen by Thomas Bresson, seta with pollen by István Mikó, *Arabidopsis* plants with hair-like structures (trichomes) by Annkatrin Rose, *Drosophila* photo by John Tann, *Drosophila* bristles redrawn from [119], scanning electron micrograph of *Arabidopsis* trichome by István Mikó, zebrafish embryos by MichianaSTEM, zebrafish blood vessels from [120]. Figure assembled by Anya Broverman-Wray.

Phenotypes, i.e., observable traits above the molecular level, such as anatomy and behavior, underlie, and indeed drive, much of the research in the life sciences. For example, they remain the primary data we use to define most species and to understand their phylogenetic history. Phenotype data are also used to recognize, define, and diagnose pathological conditions in plants, animals, and other organisms. As such, these data represent much of what we know of life and are, in fact, necessary for building a comprehensive tree of life [1]. Our observations of organismal phenotypes also inspire science aimed at understanding their development, functions, evolution, and interactions with the environment. Research in these realms, for example, has uncovered phenotypes that could be used to create antimicrobial materials [2] and efficient microrobots [3], yield novel approaches for drug delivery [4], treat the adverse effects of aging [5], and improve crop traits [6], among many other applications. Disease phenotypes, likewise, provoke us to research their genomic and environmental origins, often through manipulations of model organisms and/or by exploring the wild populations and ancestors, especially in the case of plants. The gamut of research on phenotype is very broad, but given the lack of computability across phenotype data (Fig. 1, bottom panel), there exists minimal cross-domain interaction. By not investing in the infrastructure needed to share phenotype data, we are missing opportunities for extraordinary discoveries.

Annotation strategies for genomes, in contrast to phenomes, are well advanced, with common methodologies, tools, syntaxes, and standards for articulating a precise description of nearly every type of genomic element [7]–[12]. Genomic data are also aggregated into large datasets, e.g., NCBI [7], EBI [8], DDBJ [9], and others [10]–[13]. Researchers lack these similarly well-established, linked, and consolidated resources for describing phenotypes and the contexts in which they arise, despite previous calls for more investment in this area [14]–[17]. Phenotype data (Table 1), although abundant and accumulating rapidly—e.g., species descriptions, image databases, analyses of induced variation, physiological measurements, whole genome knockout studies, high-throughput assays, electronic health records—are extremely heterogeneous, largely decentralized, and exist predominantly as free text. Thus, phenotype data are difficult to locate and impractical to interpret. In some areas of research, such as crop genetics and patient care, a great majority of the phenotype data underlying published research is not publicly available [18]. There also exists a divide between quantitative data and qualitative phenotype data, requiring reference measures or populations and statistical cutoffs to support interoperability (for example, “large head” versus a head circumference measurement). Finally, phenotypes change over time—be it evolutionary time, disease-course time, or developmental time—and the timing and ordering of phenotypic presentation is specific in any given context yet is rarely communicated. In short, while phenotype data are as complex, diverse, and nuanced as genomic data, they have not seen data standardization and analyses applied with the same broad strokes as we have seen for genomics.

**Table 1:** Finding phenotypes.

Phenotype data source	Characteristics	Example/Reference
published literature from biological and biomedical domains	highly dispersed corpus, mainly digitized, but still in natural language; contains abundant phenotypes	publisher websites, reviews that summarize important reference phenotype datasets [79],[80]
supplementary data	spreadsheets, text files	publisher repositories, open repositories (e.g., Dryad [81])
trait databases and large corpora	relational databases containing free text phenotype descriptions	phenotype repositories specific to a particular field of study [82], Biodiversity Heritage Library [83], Encyclopedia of Life [62], Plant Trait Database [84], morphology databases [85]–[87]
images	annotated with keywords (free text); dispersed across many databases and repositories; phenotype or genotype data contained in these images are not computationally accessible [78].	biodiversity image stores [85]–[89], patient MRI images, X-rays, bright-field micrographs, image-bases of plant phenotypes [90]
natural history collections	>3,000,000,000 biological specimens worldwide, some with free text descriptions and associated images	iDigBio [91]
auto-generated data	quantitative data from satellite tracking devices, environmental sensors, and high-throughput phenotyping processes	National Ecological Observatory Network (NEON) [92], high throughput [26]–[28], tracking sensors [93]

The rich legacy of research in the life sciences includes a wealth of phenotype data contained in many sources, for millions of extinct and extant species. Some important sources of phenotypes date from more than 250 years ago [74]–[77]. With very few exceptions, phenotype data are not computationally accessible [78].

Nevertheless, a small quantity of phenotype data, for a handful of species, is indeed formalized, such that it can be reliably searched, compared, and analyzed computationally (see below). However, with many disparate approaches to formalizing phenotypes, including different annotation strategies, the use of unrelated vocabularies, and the use of incomparable models and formats—these data are not fully unified or interoperable between taxa.

Given the latent potential of phenotype data and the emerging approaches to representing and computing across phenotypes, we members of the Phenotype Research Coordination Network (Phenotype RCN) [19], feel that the time is ripe for system-wide investment in the development of the needed tools and standards. As described in Box 1, many projects, sometimes working together but often independently, have begun building the foundation. There is now an opportunity for the large cross-domain phenomics research community to take advantage of new technologies for analyzing and managing the vast and diverse landscape of phenotype data, if attention and resources are applied to build in a consistent fashion on the current foundation.

**Box 1. Methodologies to Make Phenotypes Computable**

The prospects of computable phenotype data have slowly improved over the past several years, with several domain-specific initiatives yielding results [21],[30],[32],[94],[95] and a larger framework of data integration resources [96]–[100]. These pioneering projects have achieved several goals: (i) more standardized measurements of complex phenotypes (e.g., PhenX [101]); (ii) an integrative phenotype semantic representation (in Web Ontology Language [OWL] [102]) and its use [103]–[105] to capture the genetic and environmental context of an observed phenotype [106]; (iii) an ontology of classes defining the anatomical, behavioral, and biological function terms and the relevant phenotypic qualities needed to describe phenotypes effectively in detail; and (iv) algorithms, such as OWLSim [107],[108], combining the logical connections inherent in the ontologies with statistical analyses to identify phenotypes that are correlated with specific genetic makeups.

These tools have been used effectively in both the model organism biomedical and biodiversity domains, for example to discover new genes involved in gene networks underlying human disease [95],[109]–[111], to prospect for candidate genes associated with crop improvement using Genome-Wide Association Studies (GWAS) experiments [112],[113], to propose candidate genes for evolutionary novelties [21], to integrate and organize diverse functional data [114], to understand the characteristics used to diagnose species [30],[31] and, when combined with systems biology data such as protein–protein interactions or pathway resources, to augment the analysis used in a clinical setting for diagnostics [95],[115]–[117]. The use of computable phenotypes is expected to be a powerful approach to discovery of the genetic contribution to phenotypes, and it applies across all categories of genetic elements.

## Building a Phenomics Discovery Environment

How do we develop an environment in which researchers can readily make discoveries concerning the intimate connections among phenotypes, environment, and genetics? Three requirements must be met for this vision to become a reality across large-scale data. First, phenotype descriptions must be rendered in a computable format, which usually involves the use of appropriate ontology terms (via Uniform Resource Identifiers [URIs]) to represent the phenotypic descriptions found in narrative text or data sources. Each bit of text is thereby imbued with properties and relationships to other terms (Fig. 1, top panel). Second, these semantically represented phenotype data, which integrate the phenotypes (Fig. 1, top panel) across species and also with their genetic and environmental contexts, must be stored in a way that is broadly accessible on the Internet in a nonproprietary format, e.g., in a Resource Description Framework (RDF). The third requirement is to grow a set of algorithms that enable users to analyze the data. That is, these algorithms combine the logical connections inherent in the ontologies with statistical analyses to, for example, identify similar phenotypes and their correlations with specific genetic or environmental factors.

Examples of systems that have the potential to transform their fields come from several domains. For instance, by computing from natural species phenotypes to the phenotypes resulting from gene disruption in model organisms, the Phenoscape project [20] demonstrated that genes underlying evolutionarily novel phenotypes can be proposed for experimental testing [21]–[23]. Uniting these previously unlinked data from evolutionary and biomedical domains provided a way to virtually automate the formulation of evolutionary developmental (evo-devo) hypotheses. The reinvention of descriptive taxonomy as a 21st century information science, likewise, requires computable phenotypic data and resources [24], including those for taxonomy [25] and for evolutionary biology [26]–[28]. This process is an active research focus of the Hymenopteran Anatomy Ontology project [29], which is developing computational methods to allow descriptions of species' phenotypes to be made in explicit and searchable forms [30],[31]. Other successes have come from linking human disease phenotypes to annotated genetic data from model organisms, thus yielding insights into the genes involved in human disease [32],[33]. Similarly, the Gramene project [34] developed the plant Trait Ontology (TO) to annotate the Quantitative Trait Locus (QTL) [35] for several crop plants, including rice, maize, and wheat.

Remarkably, and despite their significantly different aims, much of the phenotypic data that have been amassed through these projects can be made comparable—an outcome that until recently would have been impossible—because each of these groups shared common ontologies (i.e., semantics) and data annotation strategies. The systems they used are thus logically interoperable, and the bodies of phenotypic data emerging from their work can be compared and aggregated without further intervention. For these limited and domain-specific successes to be brought to bear more generally, approaches to ontology development and data annotation must be scaled up.

Several hurdles must be overcome. First, only a small fraction of the phenotypic diversity of life is currently represented in phenotype ontologies. Ontology development is time-consuming, requires expert knowledge and community buy-in, and is ideally paired with data-driven research that iteratively checks the soundness of the ontology as it simultaneously seeks discovery. New approaches are needed to expedite ontology development. Second, current methods of phenotypic data annotation are largely manual, thus requiring substantial resources for personnel to translate data from the published literature into a computable format. Semi-automated approaches for extracting phenotypes and other data from text [36]–[38] must be further developed. Though time-consuming, the transformation of legacy data in relation to these resources should be a one-time investment. It is only possible, however, if current and future projects co-develop and adopt common standards, and actively contribute to their ongoing development and maintenance, and if researchers avoid practices that may create errors [39] by writing their descriptions in ambiguous or locally idiosyncratic ways. Thus we must involve authors, editors, publishers, and funding agencies in the entire scholarly communication process in establishing the needed resources needed for data interoperability.

Predicting an individual organism's phenotypic characteristics based on the combination of its genetic heritage, development, and environmental context is a challenge for research at the intersection of the physical and life sciences [40] and is a driving force behind a major cyberinfrastructure investment by the United States National Science Foundation (NSF) [41]. With focused attention on the requirements for a phenomics-based system, we can expedite this goal. Integrating species phenotypes with data across all levels of the biological hierarchy is possible if strategies for data management are co-developed and coordinated.

## Achieving Data Integration

**Figure 2:**
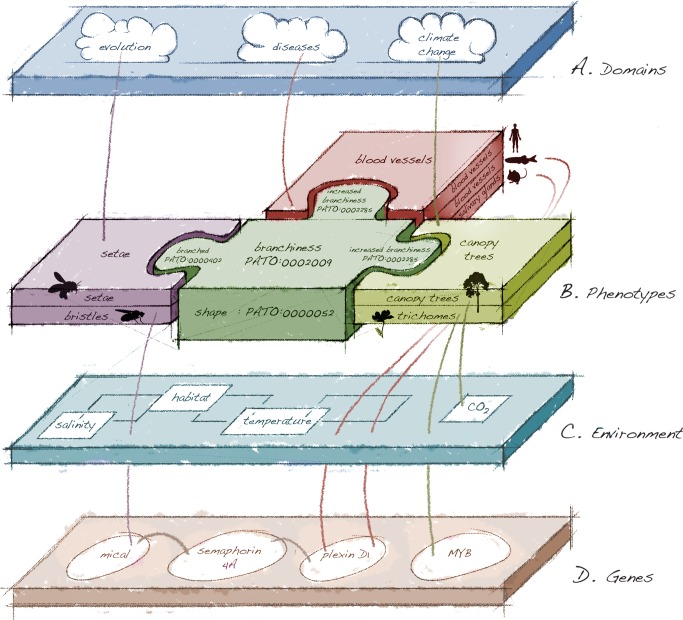
Phenotypes shared across biology. Phenotype data are relevant to many different domains, but they are currently isolated in data “silos.” Research from a broad array of seemingly disconnected domains, as outlined here, can be dramatically accelerated with a computable data store. (**A**) **Domains**: Diverse fields such as evolutionary biology, human disease and medicine, and climate change relate to phenotypes. (**B**) **Phenotypes**: insects, vertebrates, plants, and even forests all have features that are branched in some way, but they are described using different terms. For a computer to discover this, the phenotypes must be annotated with unique identifiers from ontologies that are logically linked. Under “shape” in the PATO quality ontology [106], “branchiness” is an encompassing parent term with subtypes “branched” and “increased branchiness.” From left to right, top layer, insects, vertebrates and plants have species that demonstrate phenotypes for which the genetic basis is not known. Often their companion model species, however, have experimental genetic work that is relevant to proposing candidate genes and gene networks. Insects (1): An evolutionary novelty in bees (top layer) is the presence of branched setae used for pollen collection. Nothing is known about the genetic basis of this feature. One clue to the origin of this evolutionary feature comes from studies of *Drosophila* (bottom layer), where *Mical* overexpression in unbranched wild-type bristles generates a branched morphology [119]. Mical directly links semaphorins and their plexin receptors to the precise control of actin filament dynamics [119]. Vertebrates (2): In humans, aberrant angiogenesis, including excessive blood vessel branching (top layer), is one of the six central hallmarks of cancer [121]. Candidate genes have been identified using data from model organisms. In zebrafish (middle layer), studies of the control of sprouting in blood vessel development show that signaling via semaphorins [122] and their plexin receptors is required for proper abundance and distribution [123]; disruption of *plxnd1* results in increased branching [120],[124],[125]. In mouse (bottom layer), branching of salivary glands is dependent on semaphorin signaling [126], as is the branching of various other epithelial organs [127]. Plants (3): The uppermost canopy of trees of the rainforest (top layer) undergo a marked increase in branching associated with climate change [128]. Nothing is known about the genetic basis of this feature. The branching of plant trichomes (bottom layer), tiny outgrowths with a variety of functions including seed dispersal, has been studied in the model *Arabidopsis thaliana.* Branching occurs in association with many MYB-domain genes [129], transcription factors that are found in both plants and animals [130]. (**C**) **Environment**: Diverse input from the environment influences organismal phenotype. (**D**) **Genes**: At the genetic level, previously unknown associations with various types of “branchiness” between insects and vertebrates are here made to possibly a common core or network of genes (the semaphorin-plexin signaling network). No association between genes associated with plant branching (Myb transcription factors) and animal branching is obvious from the literature. Image credit: Anya Broverman-Wray.

Researchers who attempt to explore biological data using a multidisciplinary approach are aware that it is nearly impossible to integrate comparable data from multiple species and multiple publications. We manually assemble an example (Fig. 2) of how large-scale availability of logically structured phenotype descriptions could inform and relate disparate fields of research and help address this significant problem. Past efforts, however, have largely involved manual integration of limited datasets. In the future, the study of phenotypic causality will be increasingly reliant on large and rapidly growing data stores that can only be effectively searched with automated or semi-automated methods. At this juncture, discoveries in many areas of biology rely on integrating genomic data with phenotypic data, and such integration is at an impasse because of the lack of computable and accessible phenotypic data within and across species [42].

### Linking Phenotypes to Genomic and Genetic Variation Data

Given that genomic data are now relatively inexpensive to collect (approximately US$5,000 per individual genome and rapidly approaching US$100 [43]), a growing number of independent projects are explicitly linking genetic variants to related phenotypes at costs upwards of US$1 million per species genome. For example, the NCBI databases [7],[44] capture data concerning human variants related to disease using semantic terms [45]–[47]. Large-scale integration of such variants, including computable descriptions of disease phenotypes in humans, model and non-model organisms, are collected and semantically integrated to help support disease diagnosis and mechanism discovery by the Monarch Initiative [33]. The National Institutes of Health (NIH) Undiagnosed Disease Program [48] captures individual patient phenotype profiles using the Human Phenotype Ontology (HPO) and submits these phenotype data to the database of Genotypes and Phenotypes (dbGaP) [49] and to PhenomeCentral [50] to aid patient matching based on semantic comparisons. Multiple projects and institutions have collaborated to develop an approach for the capture of standardized human pathogen and vector sequencing metadata designed to support epidemiologic and genotype–phenotype association studies [51]. The NIH Knockout Mouse Phenotyping Program (KOMP^2^) [52] and the International Mouse Phenotype Consortium (IMPC) [53] provide both their quantitative and qualitative phenotype assay data for the mouse using the Mammalian Phenotype Ontology (MP) [54]. Both HP and MP classes (i.e., descriptive terms) are linked to upper-level classes in the UBERON anatomy ontology [55],[56]. Thus, the phenotypes and associated variations from these autonomous projects can be compared automatically, as evident in cross-species resources such as PhenomeNET [57] and others [58],[59]. Similarly, the Gramene project [34] developed the plant Trait Ontology (TO) to annotate the Quantitative Trait Locus (QTL) [35] for several crop plants, including rice, maize, and wheat. As noted above, however, the paths between genotype and phenotype are not one-to-one. Any successful strategy must also account for environmental contributions, and, as with phenotypes and genotypes, a well-structured, consistent means of describing environmental differences is essential.

### Linking Phenotypes to Environment

An organism's phenotypes result from the interplay of environment with genetics and developmental processes. The meaning of “environment” differs according to biological context. For biodiversity, environment refers to the specific conditions and geographical location in which any given organism is found. For model organisms, environment comprises the experimental perturbations relative to what is “normal” for an organism of that time, for example, changes in exposure to a drug or in the concentration of salt in the water that serves as an organism's home. For epidemiological studies, environment may refer to features in the physical proximity, such as to a nuclear plant, or relate to prior personal behavior, such as a history of smoking. Although the phenotype data collected in these different types of environments may at first glance seem mutually irrelevant, there is, in fact, often a need to combine them. Exposure to an environmental toxin, for example, could similarly affect the phenotype of local flora and fauna populations and of human patients, and it could be related to phenotypic outcomes identified via experiments involving perturbation of the environments of model organisms. Neither environment nor phenotype is a static entity; both change over developmental and evolutionary time [15],[16]. Very few efforts have attempted to relate phenotypic data captured in these varied contexts, in part due to the vastly different mechanisms by which the environmental variables and measures are described.

Building blocks to capture these pieces include the Environment Ontology (EnvO) [60] and the Exposure Science Ontology (ExO) [61], which provide controlled, structured vocabularies designed to enable representation of the relationships between organisms and biological samples to their environment. EnvO has been used by projects as disparate as the Encyclopedia of Life [62] and the International Census of Marine Microbes [63]. It is also one of the ontologies incorporated into the Experimental Factor Ontology (EFO) [64] used for systematic description of experimental variables available in European Bioinformatics Institute (EBI) databases [8] and for National Human Genome Research Institute's catalog of published GWAS [65]. Ontologies and associated tools provide a powerful, rational means for discovering connections between data from multiple projects. This potential can only be realized by reusing and combining classes from core primary ontologies. This is the strategy used by numerous successful cases, such as the EFO's incorporation of EnvO and other ontologies, and has dual benefits. It allows projects to tailor their ontology to suit their own particular needs, while retaining the powerful capability to semantically integrate their data with data from multiple other projects. This approach brings convergence, avoids duplication of effort and enables joint analysis of combined data.

Remarkable advances are being made in measuring environmental data, ranging from fine-scale measurements across the surface of a leaf to variation across a planted field to high-resolution environmental layers at a global scale (e.g., [66],[67]). As environmental data rapidly accumulate as a result of these new technologies, now is an opportune moment to ensure the usability and longevity of these data by adopting systematic standards. Towards this end, recent workshops funded by NSF [68] and National Institute of Environmental Health Sciences (NIEHS) [69] brought together diverse sets of experts to aid in developing vocabularies and standards for describing environment.

## Recommendations

### Recommendation 1

We urge all biologists, data managers, and clinicians to actively support the development, evaluation, refinement, and adoption of methodologies, tools, syntaxes, and standards for capturing and computing over phenotypic data and to collaborate in bringing about a coordinated approach. And we urge university lecturers to introduce their students to these tools and concepts and integrate them into the standard basic curriculum in all relevant fields. The resultant increase in interoperability will enhance broad access to large stores of phenotypic data required or already existing across many areas of biology. It will accelerate discoveries across biological domains and increase significantly the return on the huge past and present investment made to generate the data. Although there are daunting challenges to this critical and enormous undertaking, its success will increase efficiency, greatly reduce the loss of data and duplication of effort, and facilitate reuse of phenotype data [70].

### Recommendation 2

We urge publishers to require contribution of structured phenotype data in semantic-enabled ways as the technology is developed, to enable us to compute beyond the impasse of the free-text narrative. Moreover, funding agencies should request appropriate metadata for phenotypic descriptions, and they should require that all phenotypic screening made with their funds result in open and interoperable data.

### Recommendation 3

With the community, conceptual, and methodological framework falling into place, the next steps require a new set of resources for phenotypes, including tools for the conversion of important legacy phenotype datasets to the newly established computable formats, putting into place mechanisms to scale up acquisition of new phenotypes, methods that ensure appropriate mark-up and deposition of phenotypic data upon publication [71], organization of the data into accessible online resources, new tools to visualize and analyze the data, and the development of a comprehensive cross-species and cross-domain phenotypic resource.

These needs are urgent and reach across the research spectrum, from understanding biodiversity loss and decline, to interpreting genomes of the new “non-model” systems that are coming online, to elevating the health of the expanding human population. The use of computable phenotypes is expected to be a powerful approach to discovery of the genetic contribution to phenotypes [72],[73], and it applies across all categories of genetic elements.

Science revolves around gathering facts and making theories, a repeating cycle of improvement and increasing knowledge. In the history of science, the iterative accumulation of well-integrated facts—starting with the creation of a common system of units—has over and over again determined accelerated growth in scientific understanding. As our base of phenotypic knowledge grows ever larger, it will only become ever more difficult to navigate and comprehend, without the coordinated improvements in infrastructure and culture that will expedite scientific discovery.
